# Cancer Risk Concerns and Communication Gaps Regarding GLP-1 Medications

**DOI:** 10.1001/jamanetworkopen.2025.21878

**Published:** 2025-07-18

**Authors:** Roxanna Attar-Olyaee, Ramez Kouzy, Michael K. Rooney, Zakaria El-Kouzi, Karen E. Hoffman, Sherif M. Gadoue, Comron J. Hassanzadeh, Junyi Jessy Li, Osama Mohamad

**Affiliations:** 1 Texas Tech Health Science Center El Paso, El Paso; 2 Department of Radiation Oncology, The University of Texas MD Anderson Cancer Center, Houston; 3 Department of Linguistics, University of Texas at Austin, Austin

## Abstract

This cross-sectional study analyzes social media posts to explore patient concerns and the extent of patient–clinician communication regarding cancer risks associated with glucagon-like peptide-1 (GLP-1) medications.

## Introduction

Glucagon-like peptide-1 (GLP-1) receptor agonists have recently become widely used across the US, substantially changing metabolic disease management.^1^ While regulatory agencies have reported no association between GLP-1 medications and cancer risks, concerns persist among patients, particularly among survivors of cancer and those with a family history of cancer.^2,3^ To our knowledge, how patients discuss these cancer-related risks on social media has not been extensively studied. We analyzed social media discussions to explore patient concerns and the extent of patient–clinician communication regarding cancer risks associated with GLP-1 medications.

## Methods

In this cross-sectional study, we collected posts and comments from the 5 most active GLP-1 medication–related forums on a social media website (Reddit; Advance Publications) in July 2024. This study was exempt from institutional review per Common Rule criteria (45 CFR §46) as it analyzed publicly available deidentified data. Using a systematic data collection approach, we identified relevant social media communities and extracted 410 710 unique entries using the application programming interface. Posts were filtered using predefined cancer-related keywords, resulting in 2059 relevant entries. We developed an artificial intelligence-assisted pipeline using the OpenAI GPT-4 model to extract predefined variables (eMethods in Supplement 1).^4^ The pipeline was optimized through iterative prompt engineering and validated against a standard data set of 100 entries independently annotated by 2 domain experts (R.A.O. and R.K.). This approach enabled systematic extraction of variables including cancer survivorship, risk perceptions, and physician discussions. We followed the STROBE reporting guideline. Data were analyzed using R statistical software version 4.3.0 (R Project for Statistical Computing).

## Results

Of 410 710 entries, we identified 1529 posts from 1067 unique users discussing cancer-related topics. Thyroid cancer was the most frequently mentioned cancer type (26.3% [402]), followed by breast cancer (9.5% [146]) (Table). Survivors of cancer authored 409 posts (26.7% of cancer-related posts), with 146 (35.7% of survivor posts) specifically discussing their use of GLP-1 receptor agonists for weight management after cancer treatment. Cancer risk discussions appeared in 784 posts (51.3%), yet only 293 posts (19.2%) mentioned discussing these risks with a physician. Notably, 319 posts (20.9%) expressed specific concerns about GLP-1 medications increasing cancer risk, while 100 posts (6.5%) discussed the potential of GLP-1 agonists to reduce future obesity-related cancer risk.

**Table.** Cancer-Related Topics in 1529 GLP-1 Social Media Posts

Topic	Posts, No. (%)^a^
Cancer type mentioned	
Thyroid	402 (26.3)
Breast	146 (9.5)
Gynecologic	44 (2.9)
Pancreas	25 (1.6)
Other^b^	141 (9.2)
Patient experience	
Survivors of cancer^c^	409 (26.7)
Family history of cancer	239 (15.6)
Survivors of cancer discussing weight loss	228 (14.9)
Survivors of cancer taking GLP-1	146 (9.5)
Cancer diagnosis after starting GLP-1	26 (1.7)
Risk communication and perception	
Mentioned GLP-1 and cancer risk	784 (51.3)
Concerned about cancer risk	319 (20.9)
Discussed cancer risk with physician	293 (19.2)
Actively seeking cancer risk data	106 (6.9)
Believed GLP-1 reduces cancer risk	100 (6.5)

Abbreviation: GLP-1, glucagon-like peptide 1.

^a^ Individual posts may contain multiple topics, resulting in percentages summing to >100%.

^b^ Other cancer types include gastrointestinal (48), hematologic (20), lung (13), and brain cancers (7).

^c^ Survivors were defined as any poster currently with cancer or who had cancer in the past, as indicated by the post.

## Discussion

Our analysis of cancer-related GLP-1 medication discussions found important patterns in risk perception and communication. Within these discussions, we found distinct themes around cancer survivorship, risk concerns, and health care clinician interactions. An important finding is the communication gap between patients and health care professionals. Although more than one-half of the posts involved cancer risk discussions, less than one-quarter reported discussing these concerns with their physicians. This indicates that important risk–benefit conversations may not be occurring in clinical settings, leading patients to seek information and support on social media platforms.

The engagement of survivors of cancer and individuals with a family history of cancer highlights a subgroup that may have heightened concerns due to their personal experiences. Notably, emerging studies have suggested that GLP-1 receptor agonists may be associated with lower rates of certain obesity-related cancers in patients with type 2 diabetes, although such data are still limited.^5,6^ Despite these findings, the lack of robust evidence and specific guidelines for high-risk populations is worth noting.

Study limitations include self-selection bias on social media, with users potentially representing extreme views, underrepresentation of non-English speakers and older adults, and lack of bot detection. Our artificial intelligence extraction pipeline may introduce additional biases, potentially misinterpreting nuanced content despite high validation metrics. Nevertheless, our findings reveal an important phenomenon in online GLP-1 and cancer-related discussions.

These findings underscore the need for enhanced patient-clinician communication about cancer risks associated with GLP-1 medications. While online forums provide peer support, personalized guidance from health care professionals remains essential. Future research should address information gaps in cancer-related discussions about GLP-1 medications, and health care clinicians should proactively initiate these conversations, particularly with high-risk individuals such as survivors of cancer.

## Notes

Supplement 1. **eMethods.** Data Collection, Data Preprocessing, Annotation Guideline Development, Human Annotation, and LLM Prompt Engineering and Evaluation

Supplement 2.
Data Sharing Statement
