# Supplementary material for: Cancer Risk Concerns and Communication Gaps Regarding GLP-1 Medications
Source: JAMA Netw Open. 2025 Jul 18;8(7):e2521878. doi: 10.1001/jamanetworkopen.2025.21878 (PMC12274973; doi:10.1001/jamanetworkopen.2025.21878)
Supplement: Supplement 1. — eMethods. Data Collection, Data Preprocessing, Annotation Guideline Development, Human Annotation, and LLM Prompt Engineering and Evaluation [file jamanetwopen-e2521878-s001.pdf]

Supplemental Online Content

Attar-Olyaee R, Kouzy R, Rooney MK, et al. Cancer risk concerns and communication gaps regarding GLP-1 medications. *JAMA Netw Open.* 2025;8(7):e2521878. doi:10.1001/jamanetworkopen.2025.21878

eMethods. Data Collection, Data Preprocessing, Annotation Guideline Development, Human Annotation, and LLM Prompt Engineering and Evaluation

This supplemental material has been provided by the authors to give readers additional information about their work.

© 2025 Attar-Olyaee R et al. *JAMA Network Open*.

eMethods

To effectively extract structured clinical data from Reddit discussions, we constructed a framework utilizing LLMs while also incorporating human expertise and iterative prompt refinement illustrated and explained below

1. Data Collection

Reddit was chosen as the platform to analyze as it offers topic-centered discussions via subreddits that are well-organized through threads. They are monitored with multiple moderators to maintain relevance which reduces the likelihood of irrelevant content during LLM extraction. Long-form posts without character limits allow users to express detailed concerns and experiences, maximizing the depth of patient-reported information. We collected 410,710 posts and comments from five GLP-1–related subreddits (r/Semaglutide, r/WegovyWeightLoss, r/Zepbound, r/Ozempic, and r/Mounjaro) using the Reddit API (July 2024). Subreddits were selected based on high engagement and relevance to GLP-1 discussions, maximizing the likelihood of patient-centered content.

2. Data Preprocessing

These posts were filtered using cancer-related keywords: ’cancer’, ’malignancy’, ’tumor’, ’neoplasm’, ’carcinoma’, ’sarcoma’, ’leukemia’, ’lymphoma’, ’metastasis’, ’oncology’, ’biopsy’, ’chemotherapy’, ’radiation therapy’, ’malignant’, and ’benign tumor’. After applying this filter and removing duplicates and non-English posts, the dataset was narrowed to 2,059 entries. We then performed manual and model-based relevance checks to confirm discussions actually related to cancer risk or survivorship and GLP-1 use, excluding metaphorical or irrelevant uses of "cancer" which yielded 1,529 entries for analysis.

3. Annotation Guideline Development

We created a detailed guideline defining variables to extract, including cancer survivorship, family cancer history, cancer type, risk perceptions, discussion with physicians, and diagnosis timing. The annotation guideline, data used, and code are available at https://github.com/ramezkouzy/GLP1-LLM.

4. Human Annotation

Two domain experts (a radiation oncologist and a medical student specifically briefed on the annotation guidelines) independently annotated a random sample of 100 entries. Annotations were reconciled by discussion, and a high inter-annotator agreement (Fleiss' $\kappa \geq 0.8$) was achieved across key variables, establishing a gold-standard dataset for model evaluation.

5. LLM Prompt Engineering and Evaluation

Initial zero-shot prompting with Open AI gpt-4o-mini accessed via API in August 2024 followed the human annotation guidelines and used a structured JSON output schema. Performance issues after initial prompting in nuanced variables (e.g., cancer type) led to prompt engineering using chain-of-thought prompting (encouraging model reasoning), few-shot examples to guide classification, inclusion of edge cases the model struggled with, and a temperature set to 0.0 for consistent outputs. Iterative prompt refinement dramatically improved extraction accuracy, with macro-averaged precision, recall, and F1 scores above 0.90. Baseline macro-average F1 improved from 0.80 to 0.90 after iterative improvements, and stability testing across five repeated runs showed 95% agreement, confirming model reliability for this task. Complete code, annotation guidelines, and detailed performance metrics are available at https://github.com/ramezkouzy/GLP1-LLM and https://arxiv.org/pdf/2411.17967.

© 2025 Attar-Olyaee R et al. *JAMA Network Open*.
