# Supplementary material for: Cancer Risk Concerns and Communication Gaps Regarding GLP-1 Medications
Source: JAMA Netw Open. 2025 Jul 18;8(7):e2521878. doi: 10.1001/jamanetworkopen.2025.21878 (PMC12274973; doi:10.1001/jamanetworkopen.2025.21878)
Supplement: Supplement 2. — Data Sharing Statement [file jamanetwopen-e2521878-s002.pdf]

Data Sharing Statement

Attar-Olyaee. Cancer Risk Concerns and Communication Gaps Regarding GLP-1 Medications. *JAMA Netw Open*. Published July 18, 2025. doi:10.1001/jamanetworkopen.2025.21878

Data
Data available: Yes
Data types: Data (not involving human participants)
How to access data: Data and code are available at https://github.com/ramezkouzy/GLP1-LLM
When available: With publication

Supporting Documents
Document types: Statistical/analytic code
How to access documents: Data and code are available at https://github.com/ramezkouzy/GLP1-LLM
When available: With publication

Additional Information
Who can access the data: Data is publicly available and code can be found at https://github.com/ramezkouzy/GLP1-LLM
Types of analyses: Data is available publicly for any purpose.
Mechanisms of data availability: The data and code can be accessed without investigator approval via https://github.com/ramezkouzy/GLP1-LLM. We ask that if you use the code to cite the manuscript.
